# Vasodilator-stimulated phosphoprotein (VASP), a novel target of miR-4455, promotes gastric cancer cell proliferation, migration, and invasion, through activating the PI3K/AKT signaling pathway

**DOI:** 10.1186/s12935-018-0573-4

**Published:** 2018-07-09

**Authors:** Haiqun Chen, Gang Dai, Yiting Cai, Qinhao Gong, Wei Wu, Min Gao, Zhewei Fei

**Affiliations:** 1 Department of General Surgery, The ChongMing Branch of XinHua Hospital, The Affiliated Hospital of Shanghai Jiao Tong University, Shanghai, 200240 China; 2 Department of General Surgery, XinHua Hospital, The Affiliated Hospital of Shanghai Jiao Tong University, No. 1665, Kong Jiang Road, Shanghai, 200240 China

**Keywords:** Gastric cancer, miR-4455, VASP, PI3K/AKT signaling pathway

## Abstract

**Background:**

MicroRNAs (miRNAs) are small non-coding RNAs which play important roles in the carcinogenesis of gastric cancer (GC). Expression profiling of miRNAs in paired gastric cancer and adjacent normal gastric tissues has demonstrated that miR-4455 is down-regulated in gastric cancer tissues, but its functional role in the carcinogenesis of GC had not previously been investigated.

**Aims:**

The purpose of this study was to investigate the functional and biological mechanisms of miR-4455 in the progression of GC, in vitro.

**Methods:**

Expression of miR-4455 was compared in human GC tissue samples and paired adjacent normal tissue samples. The in vitro effects of miR-4455 expression in MGC-803 cells on their proliferation, invasion, and migration were assessed by MTT assays and 5-bromo-2′-deoxyuridine staining, matrigel-invasion analysis and wound healing assays. Bioinformatics analysis (using PicTar, target scan and miRBase target) was used to identify potential targets for miR-4455, and the luciferase reporter assay, qRT-PCR and Western-blotting analyses were used to confirm VASP as the target of miR-4455. In addition, the effects of downregulation of VASP on the activation of PI3K/AKT signaling pathway were measured using Western-blot analysis.

**Results:**

The expression of miR-4455 was markedly down-regulated in gastric cancer tissues vs. adjacent normal tissues, and miR-4455 expression inhibited the proliferation, invasion and migration of MGC-803 GC cells in vitro. Luciferase reporter assays revealed that miR-4455 inhibited VASP expression by targeting the 3′-UTR sequence of VASP. Furthermore, silencing of VASP markedly inhibited the activation of the PI3K/AKT signaling pathway.

**Conclusion:**

Our results suggest that miR-4455 functions as a tumor suppressor in gastric cancer, by targeting VASP leading to activation of the PI3K/AKT signaling pathway and the inhibition of VASP mediated proliferation, migration and invasion of gastric cancer cells.

**Electronic supplementary material:**

The online version of this article (10.1186/s12935-018-0573-4) contains supplementary material, which is available to authorized users.

## Background

MicroRNAs (miRNAs) are small non-coding RNAs which have important functional roles in animals by regulating post-translational gene expression. They act through binding to the three prime untranslated region (3′-UTR) of their target mRNAs. It has been reported that microRNAs participate in the carcinogenetic processes of many cancers, including regulating the development, differentiation and proliferation of cancer cells [1–4]. Recent studies have indicated that microRNAs could be used as potential biomarkers and prognostic tools in gastric cancer (GC) [5–9], but the biological functions and molecular mechanisms of many of the miRNAs that have been implicated in GC remain poorly understood.

Gastric cancer is one of the most common human malignant cancers and remains the second most common cause of mortality in Asia [10, 11]. Over the last two decades, a number of miRNAs have been reported to be associated with GC development, including miR-146a [12], miR-204-5p [13], miR-486 [14, 15], miR-192 [15], miR-215 [15], miR-34b/c [16] and so on. Collectively, these reports suggest that miRNAs play important roles in GC development and progression, providing new avenues for GC diagnostic and therapeutic applications [17–19]. More recent studies have comprehensively investigated the effects of down-regulation of miR-4455 in a gemcitabine-resistant pancreatic cancer cell line [20] and in pandemrix-associated narcolepsy patients [21]. In a previous study, we demonstrated that miR-4455 was down-regulated in gastric cancer tissues compared with in adjacent normal tissues, but the underlying mechanisms were not elucidated. In the present study we aimed to identify the functional molecular target of miR-4455 and to uncover the possible regulatory pathway by which its effects are exerted.

## Methods

### Cell culture and clinical samples

The gastric cancer cell line MGC-803, normal gastric epithelial cell GES-1 and HEK293T cells were both purchased from the Type Culture Collection of the Chinese Academy of Sciences (Shanghai, China). Cells were maintained in Dulbecco’s Modified Eagle’s Medium–High Glucose (Hyclone, Logan, MA, USA) supplemented with 10% fetal bovine serum (Gibco, Grand Island, NY, USA).

A total of 30 paired samples of human primary GC tissues and their adjacent normal tissues were collected between 2015 and 2017 at the Affiliated Hospital of Shanghai Jiao Tong University, Shanghai, China. Tissue samples were frozen immediately after collection in liquid nitrogen until further use. Informed consent was obtained from all donor patients and the project protocols were approved by the Clinical Research Ethics Committee of Affiliated Hospital of Shanghai Jiao Tong University (Shanghai, China).

### Quantitative real-time PCR

Total RNA was extracted from tissue samples using TRIzol reagent (Invitrogen, Grand Island, NY, US) according to the manufacturer’s instructions. Following extraction, 2 μg of total RNA was used to synthesize the first-strand cDNA sequence in accordance with the Prime-Script RT reagent kit (TianGen, Beijing, China). The qRT-PCR analysis was then performed to quantify the relative mRNA expression using SYBR Mix (Invitrogen), with β-actin used as an internal control. Stem-loop qRT-PCR assays using TaqMan miRNA probes (Applied Biosystems, Waltham, MA) were performed to quantify the expression levels of the mature miRNAs in the sample; SncU6 was used as an internal control. The values of miRNA expression were expressed as 2^−∆∆Ct^.

### Plasmid construction and siRNA synthesis

The sequences of small interfering RNA (siRNA) for miR-4455 (miR-4455 inhibitor), and miR-4455 mimic or negative controls (miR-NC) were all purchased from Gene Pharma Co., Ltd. (Suzhou, China). The overexpressed and knockdown plasmids of VASP were both purchased from General Biosystems Co.m Ltd (An Hui, China). The direct target genes of miR-4455 were predicted using PicTar, TargetScan and miRbase Target software. The amplified 3′UTRs of VASP were cloned into the region directly downstream of a CMV-promoter-driven firefly luciferase cassette in a pGL3 vector (Beyotime, Shanghai, China). The mutant 3′-UTR of VASP, which carried the mutated sequence in the complementary site for the seed region of miR-4455, was constructed based on the p-GL3-VASP 3′UTR-wt plasmid by overlap-extension PCR.

### Cell proliferation and cell apoptosis assay

MGC-803 cells were plated onto 96-well tissue culture plates at a density of 5000 cells per well in 100 µL of DMEM high glucose medium. After transfection with miR-4455 mimic, miR-4455 inhibitor, miR-NC controls or shVASP, VASP overexpression plasmid for 48 h, cell viability was assessed using the MTT assay. Into each well was added 10 µL of 5 mg/mL MTT (Sigma-Aldrich, St. Louis, MO) and the plates were incubated for 4 h. The MTT solution was then removed and diethyl sulfoxide (Sigma-Aldrich) was added to each well to dissolve the metabolic product. Absorbance was measured at 490 nm using Multiskan FC (Thermo Fisher Scientific, Waltham, MA).

For the cell apoptosis assay, MGC-803 cells were seeded onto the wells of 6-well plates and, 48 h after transfection with miR-4455 mimic, miR-4455 inhibitor or, miR-NC controls, the cells were collected and detected using a cell apoptosis kit (BD Biosciences, San Jose, CA) and a Flow Cytometer (Beckman Coulter, Brea, CA).

### Cell invasion assays

Twenty-four well plates (8 μm pore size; BD biosciences) were coated with matrigel (BD biosciences). Cells transfected with miR-4455 mimic, miR-4455 inhibitor, miR-NC controls, shVASP or VASP overexpression plasmid were cultured overnight in serum-free medium before being trypsinized and resuspended at a density of 2 × 10^5^ cells/mL in medium containing 2% FBS. The cells were then added to the upper chamber of the well, whilst medium containing 20% FBS as a chemoattractant was added to the lower chamber. The Matrigel and the cells remaining in the upper chamber were removed by cotton swabs following 24 h at 37 °C. The cells on the lower surface of the membrane were stained with crystal violet after being fixed with formaldehyde solution. The cells in at least five random microscopic fields (× 200) were then counted and photographed.

### Wound healing assays

MGC-803 cells were seeded and plated on to six-well plates at a density of 5 × 10^4^ and incubated for 24 h. The cells were then transfected with miR-4455 mimic, miR-4455 inhibitor, miR-NC controls, shVASP or VASP overexpression plasmid for a further 24 h, after which the cells were wounded by scratching with a pipette tip and then incubated with DMEM medium containing 0.5% FBS at 37 °C for 24 h. Cells at 0 and 24 h were both photographed using a phase-contrast microscope (100×) as described by Lu et al. [22].

### Luciferase assay

Approximately 5000 cells per well were plated onto 96-well plates and co-transfected with 50 nmol/L of miR-4455 mimic or miR-NC controls, 50 ng of the luciferase reporter, and 10 ng of the pRL-CMV Renilla luciferase reporter using 0.5 μL Lipofectamine 2000 (Invitrogen). After 48 h of transfection, cells were seeded and luciferase activity was quantified using a dual-luciferase reporter assay (Promega, Madison, WI).

### Western blotting

Transfected MGC-803 cells were seeded using RIPA lysis Buffer (Beyotime, China) at 4 °C for 30 min and centrifuged at 12,000×*g* for 5 min. Concentration of proteins was measured using BCA assay kit (Beyotime). Total 40 μg protein sample was separated by 12% SDS-PAGE gel and then transferred to PVDF membranes (Millipore, Billerica, MA). Membranes were blocked with 5% non-fat milk and incubated overnight with either a rabbit anti-VASP polyclonal antibody (Abcam, Cambridge, MA, US) at a dilution of 1:1000, or with a mouse anti-β-actin monoclonal antibody (HuaAn, China) at a dilution of 1:8000. The membranes were then incubated with a goat anti-mouse horseradish peroxidase or with goat anti-rabbit horseradish peroxidase secondary antibody (Santa Cruz Biotechnology, Dallas, TX) for 2 h at room temperature as appropriate. Protein complexes were detected using enhanced chemiluminescence reagents (Invitrogen) and X-ray film, the band density was qualified by Image J software.

### Detection of bromodeoxyuridine incorporation

Untreated cells or cells transfected with siRNA or plasmid were washed thoroughly with medium and cultured in fresh medium containing 10 µM bromodeoxyuridine (BrdU; Sigma-Aldrich) at 37 °C for 1 h. Then the cells were washed with PBS, fixed in 70% ice-cold ethanol. Fixed cells were treated with 2N HCl and incubated for 30 min at RT. After washing with PBS, cells were hybridized with a mouse monoclonal anti-BrdU antibody (dilution 1:100, Abcam, USA) overnight at 4 °C. Cells were then rinsed with PBST and incubated with FITC-conjugated rabbit anti-mouse immunoglobulin antibody (Jackson Immuno Research, Lancaster, PA, USA) diluted at a ratio 1:400 in PBST. After 2 h incubation at RT in the dark, cells were washed with PBS and stained with DAPI solution for 10 min before taking photos.

### Statistical analysis

Results are presented as mean ± standard error (SEM). Data were analyzed using the two-tailed Student T test to identify differences between pairs, or by analysis of variance (ANOVA) to identify differences between more than two groups, using SPSS 17.0 (Chicago, IL). A value of *P *< 0.05 was considered to be statistically significant.

## Results

### miR-4455 is down regulated in GC tissues and GC cells

**Fig. 1:**
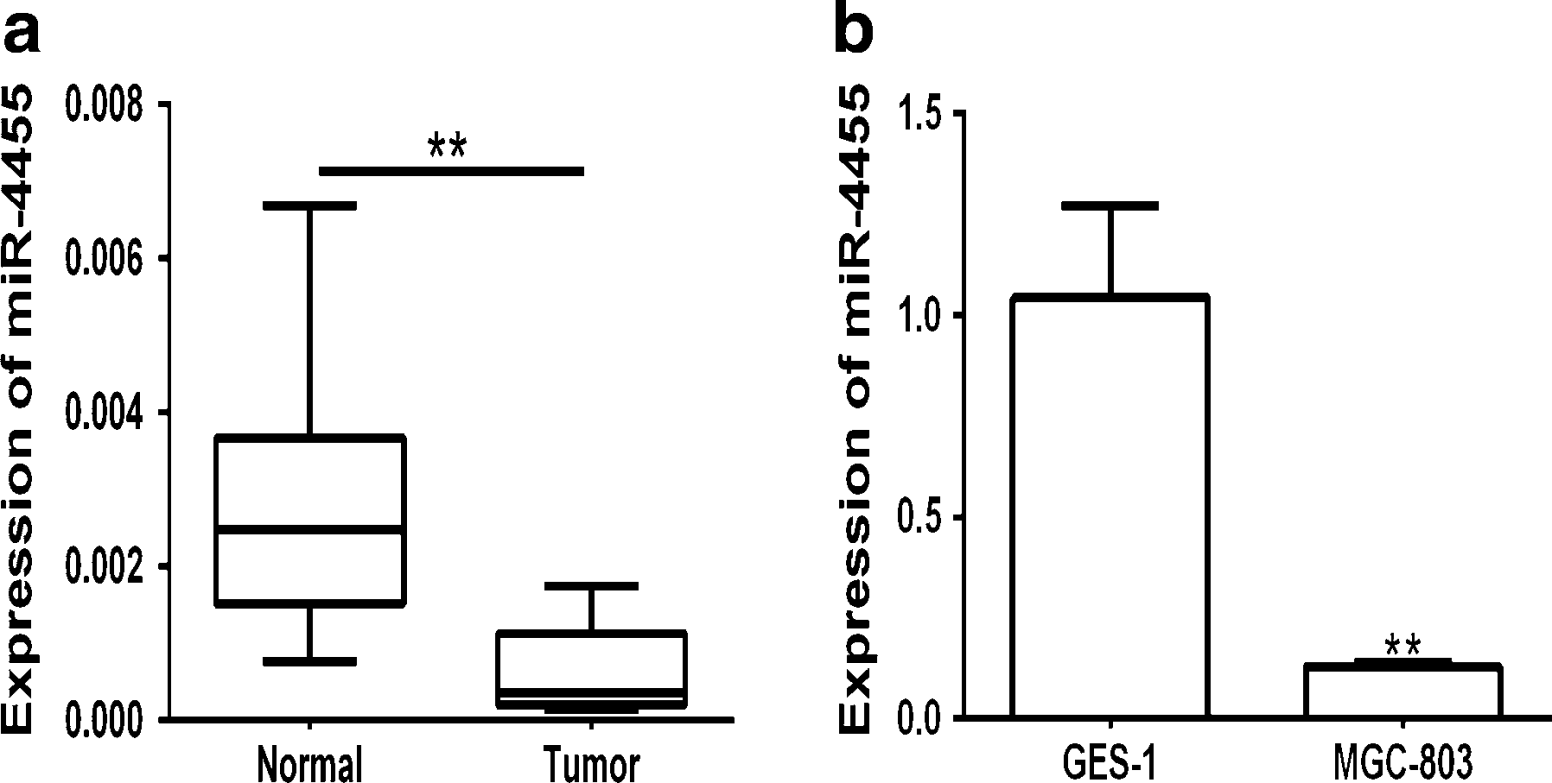
The expression of miR-4455 is down-regulated in GC tissues and GC cells. **a** Expression of miR-4455 was determined by qRT-PCR analysis in 30 pairs of human primary gastric cancer tissues (‘Tumor’) and adjacent normal gastric tissues (‘Normal’). *P *< 0.01, vs. ‘Normal’ group. **b** Expression of miR-4455 was determined by qRT-PCR analysis in normal gastric epithelial cells and gastric cancer cell MGC-803 cells. *P *< 0.01, vs. ‘GES-1’ group

Real-time RT-PCR (qRT-PCR) analysis of extracted RNA from the human paired tissue samples revealed that the expression of miR-4455 was significantly down-regulated in gastric cancer tissues compared with adjacent-normal gastric tissues (*P *< 0.05) (Fig. 1a). Also the expression of miR-4455 in gastric cancer MGC-803 cells was decreased compared with normal epithelial cell GES-1. This suggested that miR-4455 may act as a tumor suppressor in gastric cancer, the mechanism of which maybe similar to that previously described for miR-200c [23] and miR-126 [24] in GC.

### miR-4455 inhibits proliferation of GC in vitro

**Fig. 2:**
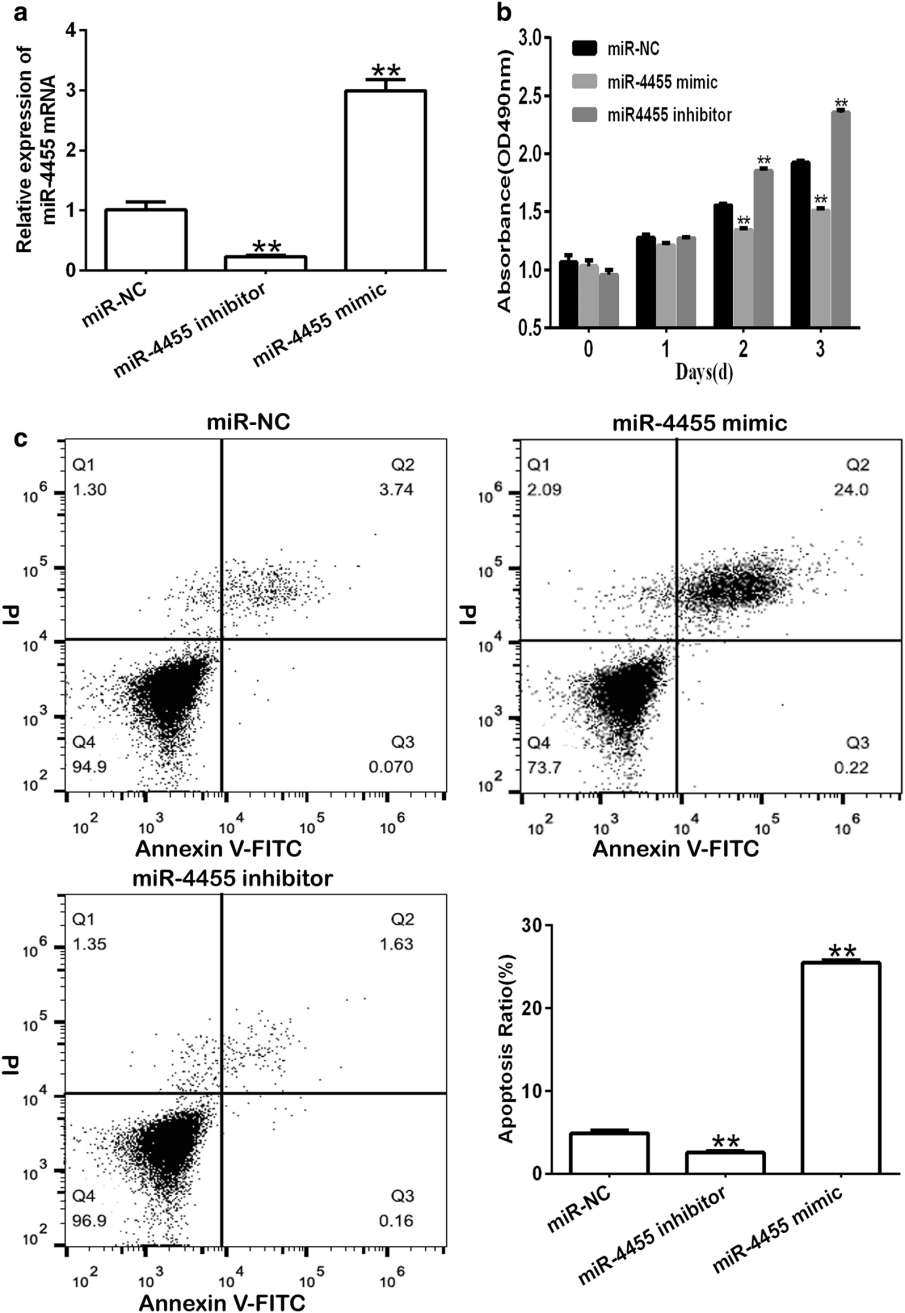
Gastric cancer cell proliferation is inhibited by miR-4455 in vitro. **a** qRT-PCR analysis of the expression of miR-4455 in MGC-803 cancer cells transfected with miR-NC, miR-4455 mimic or miR-4455 inhibitor. **b** MTT analysis of proliferation among MGC-803 cells transfected with miR-4455 mimic, miR-4455 inhibitor or miR-NC. **c** Annexin V-FITC/PI analysis of apoptosis in MGC-803 cells transfected with miR-NC, miR-4455 mimic or miR-4455 inhibitor. **P < 0.01, vs. miR-NC group

To further investigate whether miR-4455 could affect the proliferation of GC cells in vitro, we transfected MCG-803 cells with synthesized miR-4455 mimic, miR-4455 inhibitor and negative controls (miR-NC), and used qRT-PCR analysis to check the expression of miR-4455, the expression of miR-4455 was decreased in miR-4455 inhibitor but increased in miR-4455 mimic compared with miR-NC controls (Fig. 2a). We then employed an MTT assay and bromodeoxyuridine incorporation staining to measure cell viability, and annexin-V/PI flow cytometry analysis to measure the cell apoptosis ratio among the transfected cells. The results confirmed that the expression of miR-4455 was higher in GC cells that had been transfected with miR-4455 mimic compared with cells transfected with miR-NC. In addition, the proliferation of GC cells transfected with miR-4455 mimic was decreased compared with the control group (Fig. 2b and Additional file 1: Figure S1). Furthermore, miR-4455 significantly increased the cell apoptosis ratio compared with the miR-NC group (Fig. 2c).

### miR-4455 inhibits invasion and migration of GC cells in vitro

**Fig. 3:**
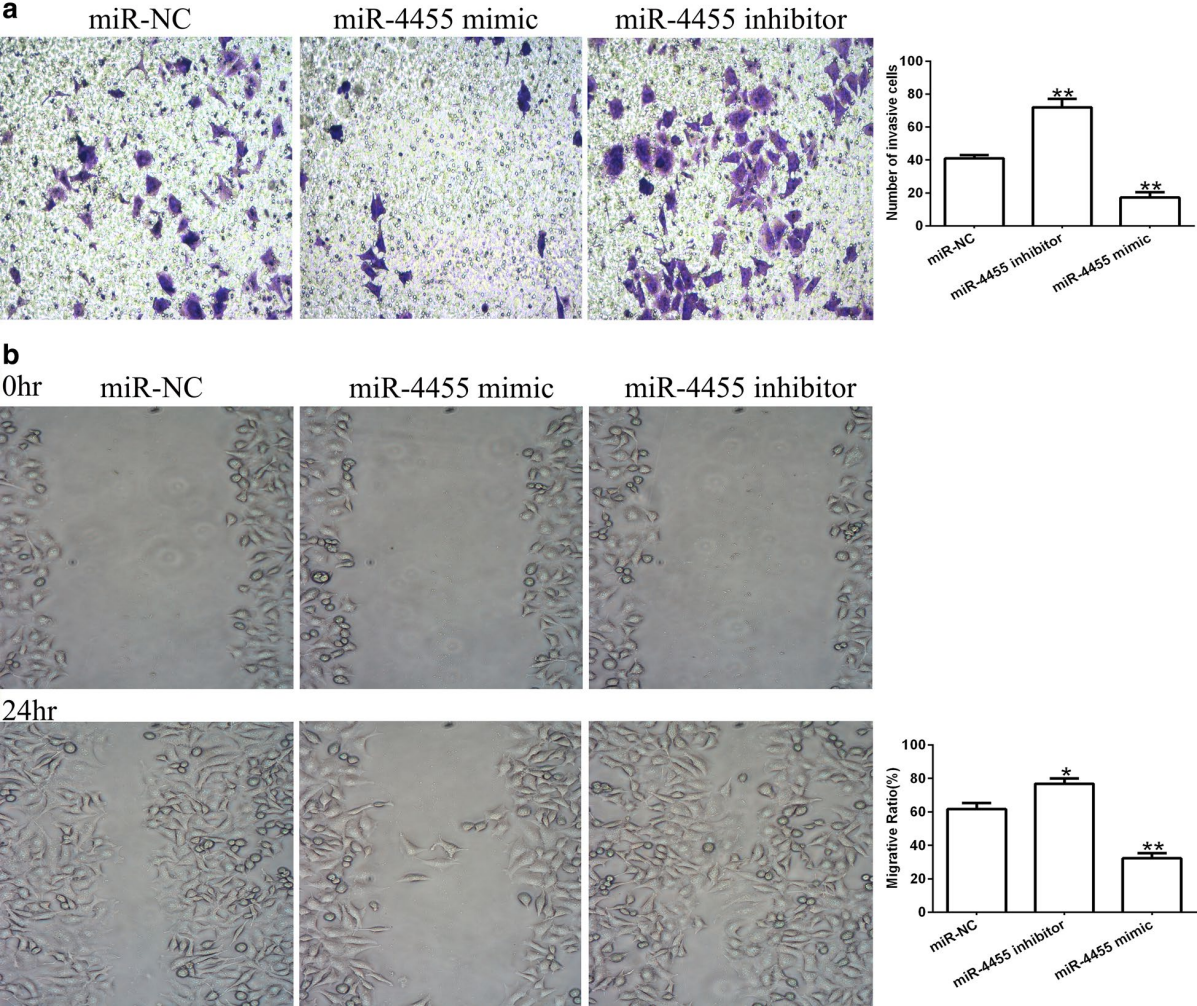
miR-4455 expression reduces the number of invasive and migrated GC cells in vitro. **a** Representative images of invasive MGC-803 cells transfected with miR-NC, miR-4455 mimic or miR-4455 inhibitor (×200), and number of invasive cells in five random fields were counted. **b** Representative images of migrated MGC-803 cells transfected with miR-NC, miR-4455 mimic or miR-4455 inhibitor (×100). The ratio of migratory and non-migratory cells among five fields were determined using Image J software. **P < 0.01, vs. miR-NC group

Based on our findings that miR-4455 could inhibit the proliferation of MGC-803 cells, we next investigated whether miR-4455 could inhibit gastric cancer cell invasion and migration processes. The results revealed that the numbers of invasive MGC-803 cells that had been transfected with miR-4455 mimic were significantly reduced compared to those of the miR-NC groups, and the migrative ratio of the cells transfected with miR-4455 mimic was also lower (Fig. 3a, b). These results suggest that miR-4455 is crucial for the migratory and invasive capability of MGC-803 cells in vitro, but the mechanism of these effects remains unclear.

### miR-4455 directly targets the 3′-UTR of VASP and suppressed its expression

**Fig. 4:**
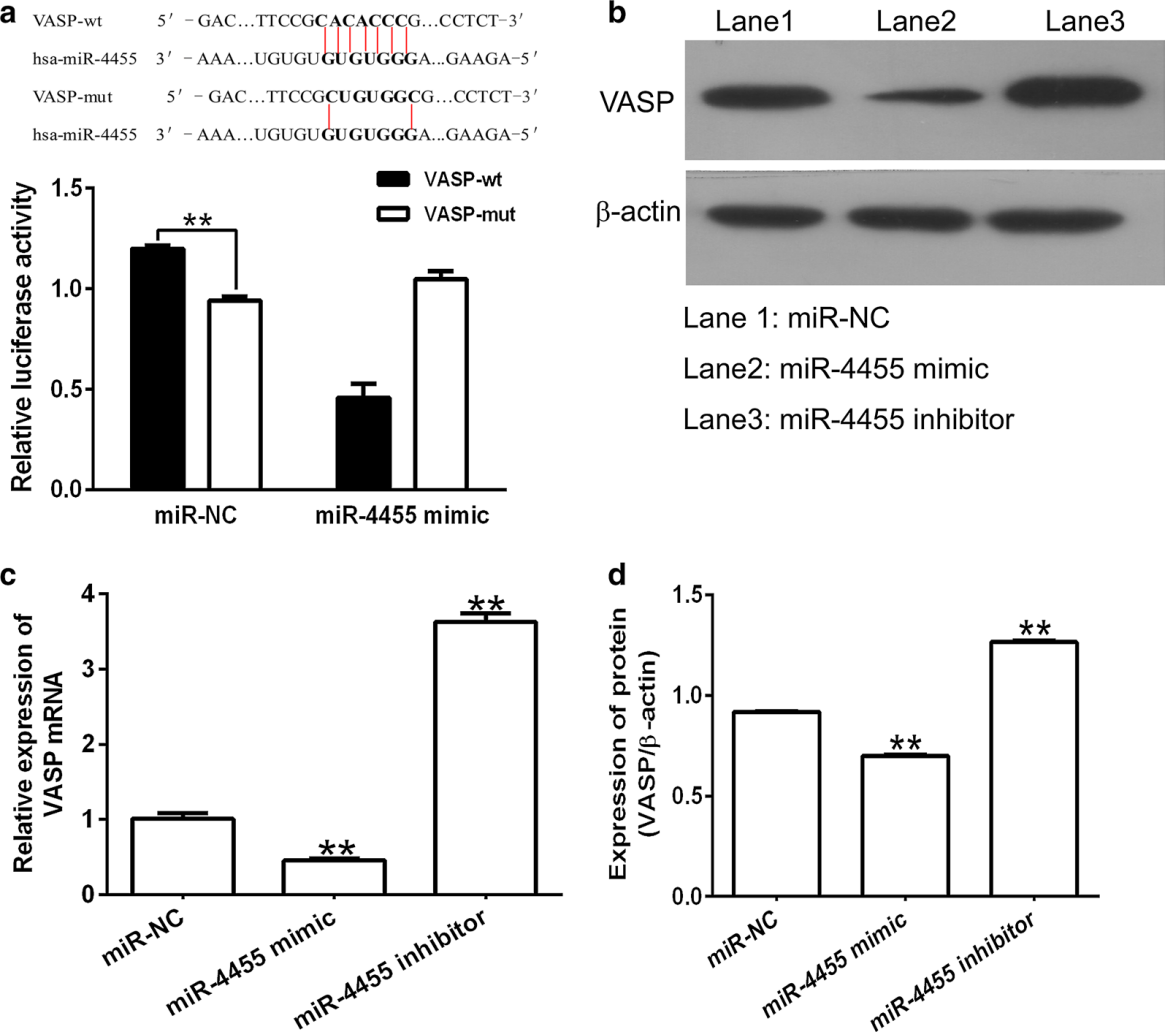
Identification of VASP as the molecular target of miR-4455. **a** Schematic of wild-type and mutant-type 3′-UTRs of the VASP plasmids, and relative luciferase activity assays of luciferase reporter plasmids containing VASP-wt or VASP-mut 3′-UTR performed in MGC-803 cancer cells. **c** The mRNA levels of VASP in MGC-803 cells cells transfected with miR-4455 mimic, miR-4455 inhibitor or miR-NC, as determined by qRT-PCR. Beta-actin served as an internal control. **b**, **d** Protein levels of VASP in MGC-803 cells transfected with miR-4455 mimic, miR-4455 inhibitor or miR-NC, as determined by Western-blot analysis. Beta-actin served as an internal control. **P < 0.01, vs. miR-NC group

The direct target genes of miR-4455 were predicted using PicTar, TargetScan and miRbase Target software. As shown in Fig. 4a, the 3′-UTR sequence of VASP is highly complementary to the seed sequence of miR-4455, indicating that miR-4455 could directly target this site.

To investigate whether miR-4455 directly recognizes the 3′-UTR sequence of VASP, we transfected the wild-type VASP-3′-UTR construct into HEK293T cells in combination with miR-4455 mimic or miR-NC. As shown in Fig. 4a, transfection with miR-4455 mimic led to a reduction in the luciferase activity of the VASP-3′UTR construct compared to the control group. In addition, after the conserved targeting regions for miR-4455 recognition were mutated, the relative luciferase activity of the reporter gene was found to be restored. We next determined whether miR-4455 could regulate VASP both at the mRNA and protein levels. The results are shown in Fig. 4b–d, which demonstrated that miR-4455 significantly down-regulated both VASP mRNA and protein levels in MGC-803 cells. These data indicate that VASP is a target gene of miR-4455 in gastric cancer cells.

### VASP silencing inhibits the activation of the PI3K/AKT signaling pathway

**Fig. 5:**
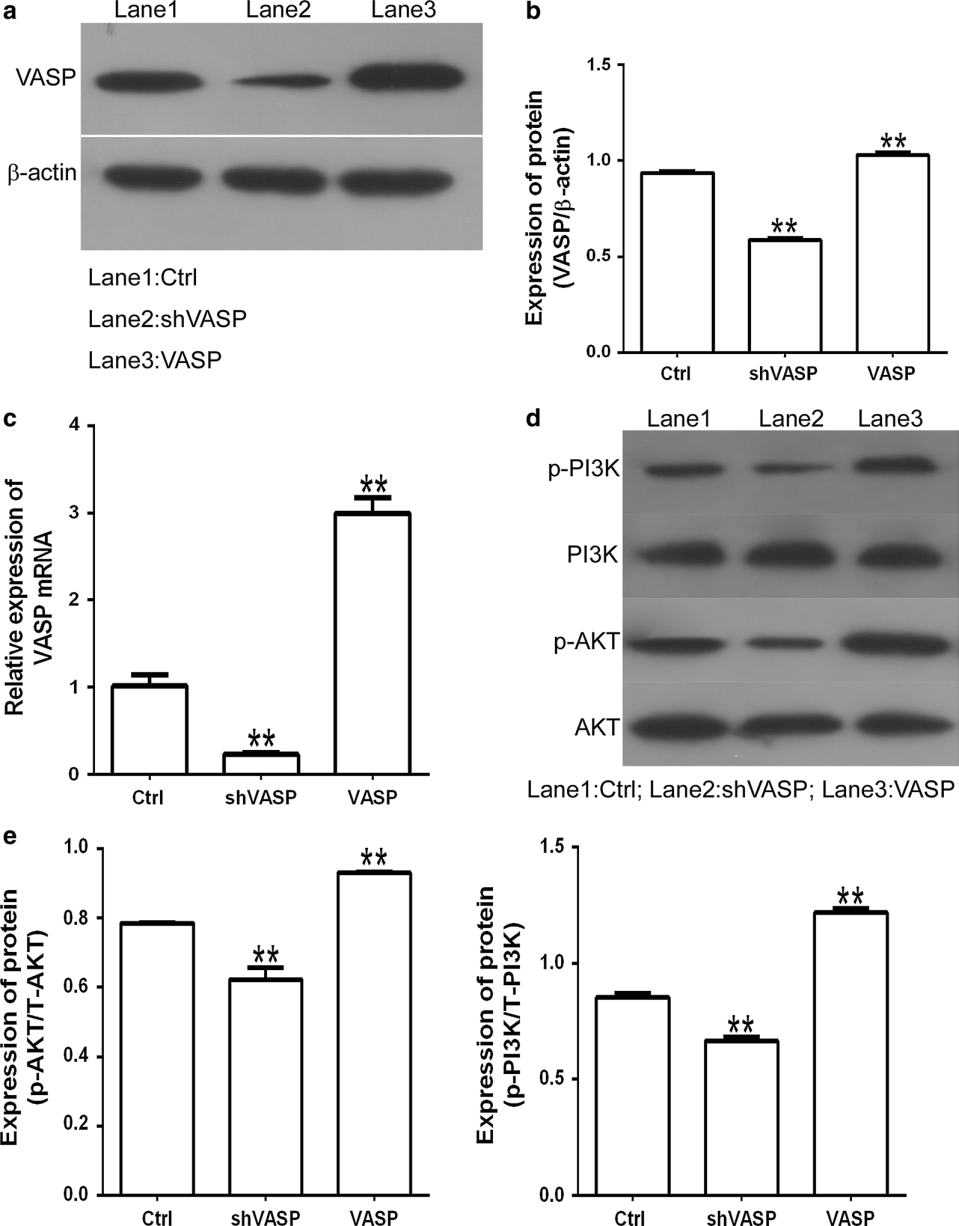
VASP promotes GC cell proliferation by regulating the PI3K/AKT signaling pathway. **a**, **b** Protein levels of VASP in MGC-803 cells transfected with VASP knockdown plasmid (shVASP), VASP overexpressed plasmid (VASP) or pcDNA3.1 negative controls (Ctrl), as determined by Western-blot. **c** The mRNA levels of VASP in MGC-803 cells transfected with VASP knockdown plasmid (shVASP), VASP overexpressed plasmid (VASP) or pcDNA3.1 negative controls (Ctrl), as determined by qRT-PCR. **d**–**f** Activation of the PI3K/AKT signaling pathway in MGC-803 cells after transfection with VASP knockdown plasmid (shVASP), VASP overexpressed plasmid (VASP) or negative control plasmid (Ctrl). **P < 0.01, vs. Ctrl groups

Existing research has suggested that VASP plays an important role in regulating the proliferation, invasion and migration of gastric cancer cells [25], but the mechanism of these effects remains unclear. To address this, we downregulated the expression of VASP in GC cells by transfection with VASP knockdown plasmid (shVASP), and subsequently detected the phosphorylation levels of the PI3K/AKT signaling pathway. The results showed that the expression of VASP was increased when transfected with VASP overexpression plasmid (VASP) compared with that of cells transfected with pcDNA3.1 control plasmid (Ctrl) (Fig. 5a–c). Furthermore, Western-blot analysis revealed that phosphorylation levels of both PI3K and AKT were significantly reduced in GC cells that had been transfected with shVASP compared with controls, indicating that VASP silencing activated the PI3K/AKT signaling pathway. Collectively, these results suggest that the regulation of gastric cancer cell invasion and migration by VASP may occur through its activation of the PI3K/AKT signaling pathway (Fig. 5d–f), in a similar manner to that previously described as PTEN and HADH molecules in GC [26, 27].

### VASP silencing inhibits proliferation, invasion and migration of GC cells in vitro

**Fig. 6:**
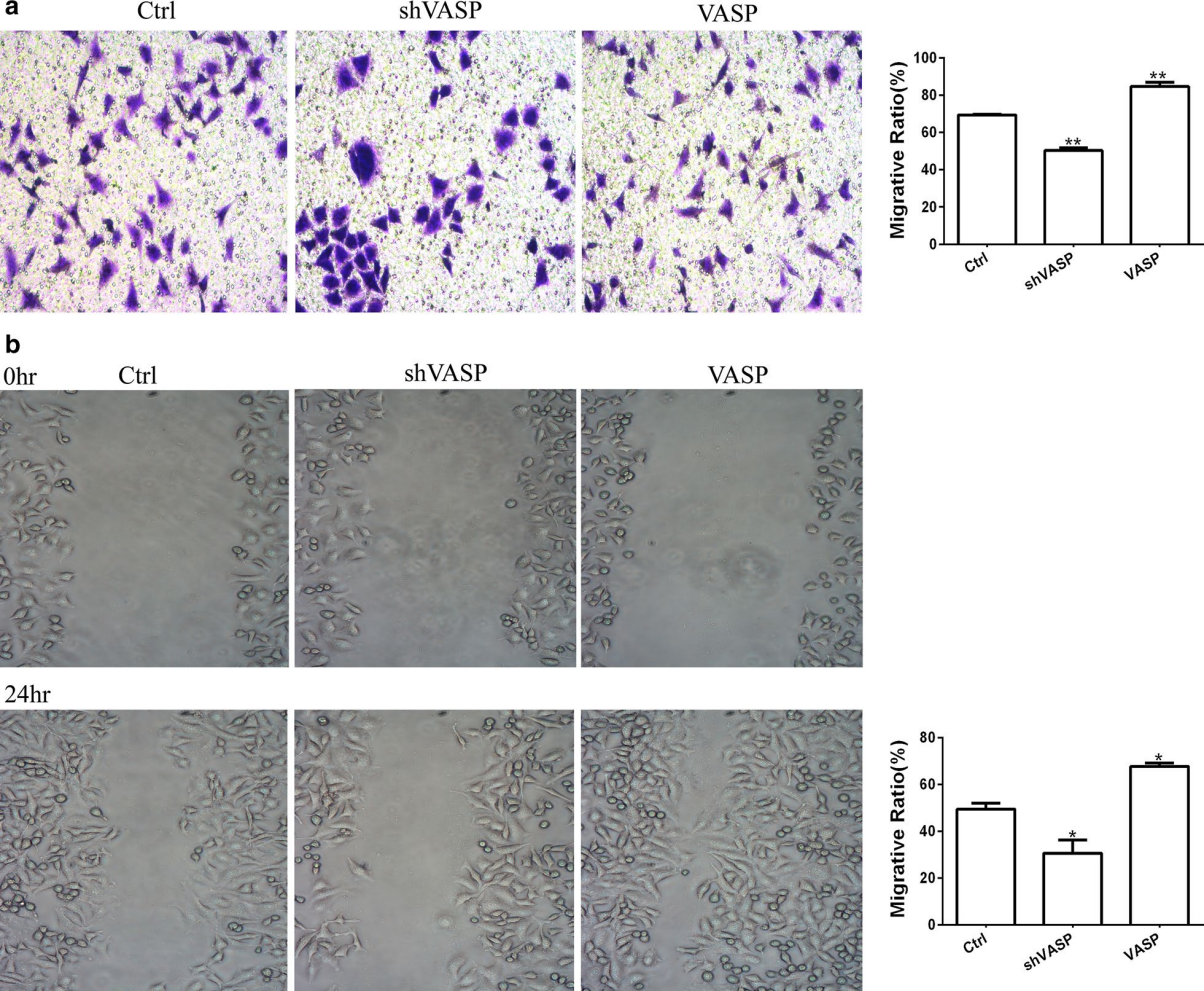
VASP silencing reduces the number of invasive and migrated GC cells in vitro. **a** Representative images of invasive MGC-803 cells transfected with negative control plasmid (Ctrl), VASP knockdown plasmid (shVASP) or VASP overexpressed plasmid (VASP) (×200), and number of invasive cells in five random fields were counted. **b** Representative images of migrated MGC-803 cells transfected with negative control plasmid (Ctrl), VASP knockdown plasmid (shVASP) or VASP overexpressed plasmid (VASP) (×100). The ratios of migratory and non-migratory cells among five fields were determined using Image J software. **P < 0.01, vs. miR-NC group

To investigate whether VASP could affect the proliferation, invasion or migration of GC cells in vitro, we transfected MCG-803 cells with synthesized negative controls (Ctrl), shVASP or VASP overexpression plasmid (VASP). We then employed an MTT assay and bromodeoxyuridine incorporation staining to measure cell viability, matrigel-transwell analysis or cell wound scratch analysis to measure the cell invasion or migration between the untreated and transfected cells. As shown in Fig. 6, the results revealed that the numbers of invasive MGC-803 cells that had been transfected with shVASP were significantly reduced compared to those of the Ctrl groups, and the migrative ratio of the cells transfected with shVASP was also lower. These results suggested that VASP is crucial for the proliferation, migratory and invasive capability of MGC-803 cells in vitro, but the mechanism of these effects remains unclear.

### Overexpression of VASP partially reversed inhibitory effects of miR-4455 on GC cell proliferation and invasion

**Fig. 7:**
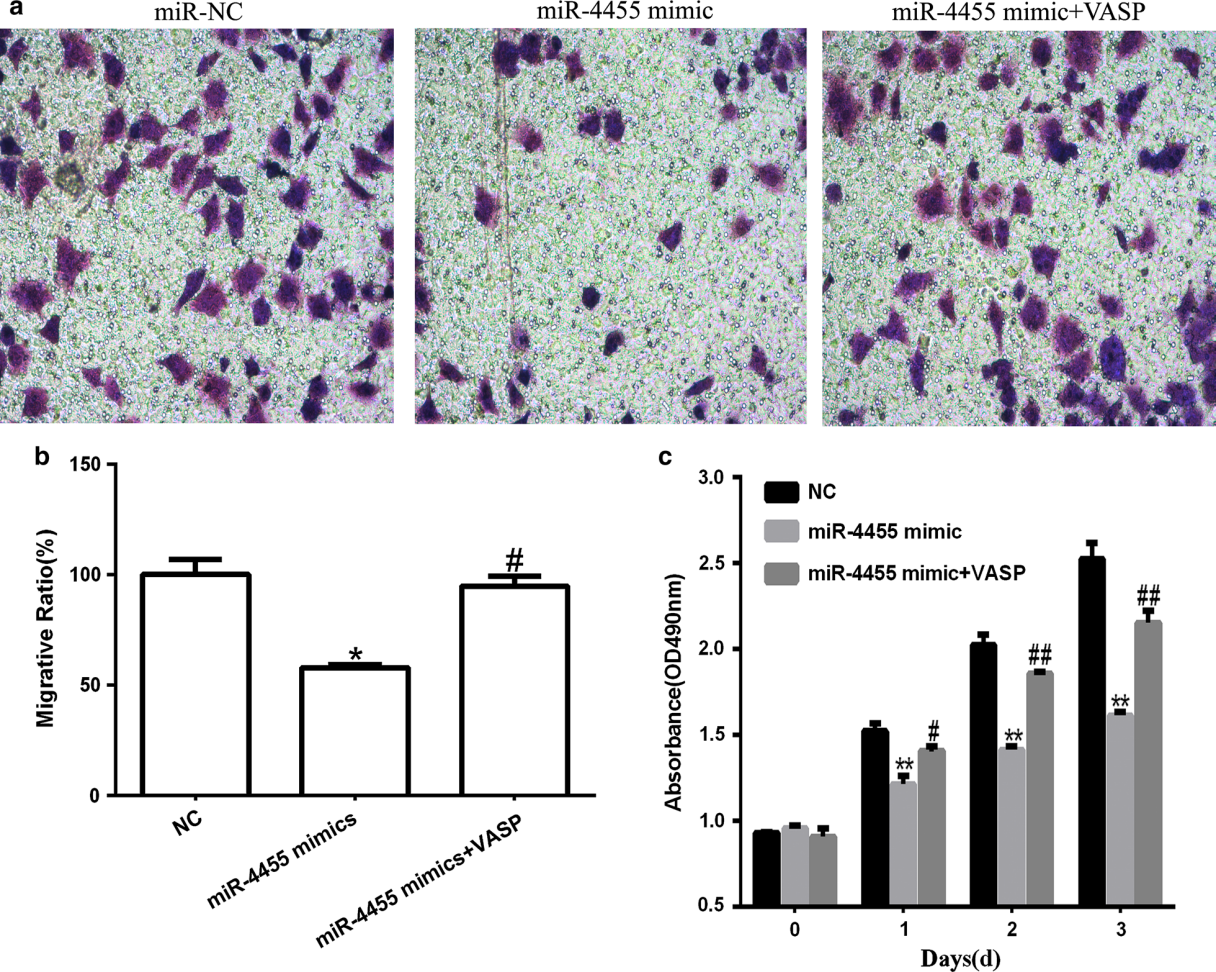
VASP attenuated the suppressive effect of miR-4455 in MGC-803 proliferation and invasion. **a**, **b** Transwell assay the invasion of MGC-803 in negative control, miR-4455 mimic or miR-4455 mimic and VASP co-transfection group (×200). **c** MTT assay the invasion of MGC-803 in negative control, miR-4455 mimic or miR-4455 mimic and VASP co-transfection group. *P < 0.05 and **P < 0.01, vs. Ctrl group; ^#^*P *< 0.05 and ^##^*P *< 0.01, vs. miR-4455 mimic group

Finally we used MGC-803 cells transfected with miR-4455 to investigate whether overexpression of VASP could reverse the inhibitory effects of miR-4455 on proliferation and invasion of MGC-803 cell. As shown in Fig. 7a, b, overexpression of VASP partially rescued proliferation and invasion capacity of MGC-803 cell transfected with miR-4455.

## Discussion

As one of the most prevalent cancers in the world, gastric cancer is also one of the commonest causes of cancer-related deaths; the 5-year survival ratio of patients with gastric cancer ranges from 10 to 30% [28]. Whilst clinical management of the disease has improved, the treatment of GC remains a significant challenge because of the complexity of its pathogenesis and the low rates of early diagnosis [29, 30]. Understanding the mechanisms involved in tumorgenesis in GC may enable its diagnosis at an earlier stage which would open up possibilities for earlier treatment. The pathogenesis of GC is complex, involving multiple stages and multiple influencing factors. A variety of genes are known to be involved, including tumor suppressor genes and oncogenes [31–35].

Existing research has revealed that the actin cytoskeleton mediates numerous cellular processes, including cell adhesion, invasion and proliferation [36]. A dysfunctional actin cytoskeleton is known to be associated with diseases such as metastatic cancers [37, 38]. As a key regulatory of the actin cytoskeleton, VASP is a member of the Ena/VASP protein family which is recruited downstream of plasma membrane receptors [39, 40] and plays a crucial role in the regulation of cell adhesion, migration and cell-to-cell interactions [41].

Recently, miRNAs have emerged as novel regulatory of the actin cytoskeleton, via the regulation of cell growth and motility [42, 43]. For example, miR-146a targets ROCK1 which affects cell movement in androgen-independent prostate cancer [44]; miR-155 is associated with TGF-induced RhoA suppression, leading to the dissolution of tight junctions between cells [45]. In the present study, we firstly found that miR-4455 was downregulated in gastric cancer tissues compared with adjacent non-tumorous gastric tissues. Next, predictions made by three bioinformatics web servers indicated that miR-4455 may be directly recognized by VASP. To test this, we used multiple transfection assays to confirm that VASP expression is regulated by miR-4455; Overexpression of miR-4455 in gastric cancer cells resulted in a significant downregulation of VASP expression at both the protein and mRNA levels. In addition, mutation of the seed sequence for miR-4455 in the 3′-UTR of VASP abrogated miR-4455 mediated inhibition of luciferase activity, clearly demonstrating that miR-4455 directly targets VASP and represses its expression. Also overexpression of VASP partially reversed the regulatory effects of miR-4455 on proliferation and invasion. Based on these results, we sought to characterize the functional significance of the regulation of VASP expression by miR-4455, and found that inhibition of VASP expression resulted in significant loss in the number of invasive gastric cancer cells. Furthermore, we showed that VASP knockdown activated the PI3K/AKT signaling pathway and decreased phosphorylation levels of PI3K/AKT proteins.

## Conclusions

In conclusion, our data demonstrate that miR-4455 suppresses tumorigenesis in GC cells in vitro, by directly targeting VASP in GC cells, finally leading to the inhibition of VASP mediated proliferation, migration and invasion of gastric cancer cells. The results indicate that miR-4455 is a novel regulatory miRNA that targets VASP in gastric cancer cells, and suggest that the miR-4455-VASP-PI3K/AKT axis offers potential as a novel therapeutic target for the inhibition of gastric cancer progression and metastasis.

## Additional file

**Additional file 1: Figure S1.** miR-4455 or VASP silencing decreased number of BrdU positive cells. (A) Immunofluorescence images of BrdU and DAPI in MGC-803 cells transfected with miR-4455 mimic, miR-4455 inhibitor or negative controls. **P < 0.01, vs. miR-NC group (B) Immunofluorescence images of BrdU and DAPI in MGC-803 cells transfected with shVASP, VASP overexpressed plasmid or negative controls The ratio of BrdU positive cells were analyzed with Image J software. **P < 0.01, vs. Ctrl group.

## Abbreviations

miRNAs: microRNAs
3′UTR: 3′untranslated region
GC: gastric cancer
DMSO: dimethyl sulfoxide
VASP: vasodilator-stimulated phosphoprotein
FBS: fetal bovine serum
BSA: bovine serum albumin
PVDF: polyvinylidene fluoride
