# Supplementary figures and images for: Vasodilator-stimulated phosphoprotein (VASP), a novel target of miR-4455, promotes gastric cancer cell proliferation, migration, and invasion, through activating the PI3K/AKT signaling pathway
Source: Cancer Cell Int. 2018 Jul 9;18:97. doi: 10.1186/s12935-018-0573-4 (PMC6038240; doi:10.1186/s12935-018-0573-4)

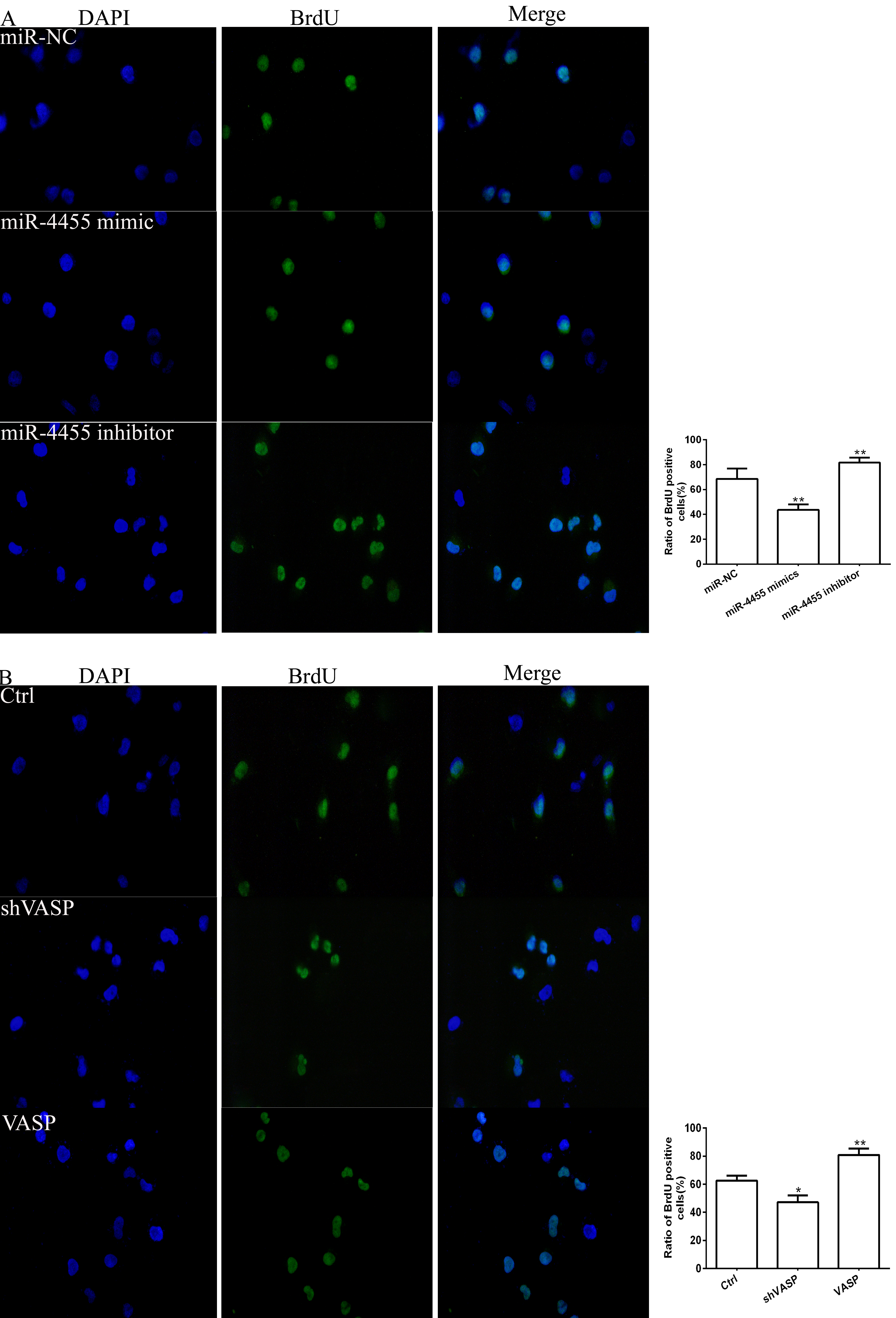

Supplement: Supplementary file 1 — Additional file 1: Figure S1. miR-4455 or VASP silencing decreased number of BrdU positive cells. (A) Immunofluorescence images of BrdU and DAPI in MGC-803 cells transfected with miR-4455 mimic, miR-4455 inhibitor or negative controls. **P < 0.01, vs. miR-NC group (B) Immunofluorescence images of BrdU and DAPI in MGC-803 cells transfected with shVASP, VASP overexpressed plasmid or negative controls The ratio of BrdU positive cells were analyzed with Image J software. **P < 0.01, vs. Ctrl group. [file 12935_2018_573_MOESM1_ESM.tif]
